# Evaluation of visual acuity in dry AMD patients after microcurrent electrical stimulation

**DOI:** 10.1186/s40942-023-00471-y

**Published:** 2023-06-18

**Authors:** Kevin M. Parkinson, Eric C. Sayre, Sheldon W. Tobe

**Affiliations:** 1 Coquitlam, Canada; 2 Vancouver, Canada; 3 Toronto, Canada

**Keywords:** Macular degeneration, Microcurrent, Retina, Visual loss

## Abstract

**Background:**

To assess micro current to improve vision for dry age-related macular degeneration. Dry age-related macular degeneration is a major cause of blindness, disability, and severe erosion of quality of life, throughout the world. Beyond nutritional supplementation, there is no approved therapy.

**Methods:**

This was a prospective randomized sham controlled clinical trial for participants with confirmed dry AMD with documented visual loss. Participants were randomized three to one, to receive transpalpebral external micro current electrical stimulation with the MacuMira device. The Treatment group received four treatments in the first two weeks, and two further treatments at weeks 14 and 26. Differences in BCVA and contrast sensitivity (CS) were estimated with mixed-effects repeated measures analysis of variance.

**Results:**

Change of visual acuity with ETDRS assessment of number of letters read (NLR) and contrast sensitivity at week 4 and 30, compared to the first visit, between 43 treatment and 19 sham control participants. The Sham Control group had NLR of 24.2 (SD 7.1) at baseline, 24.2 (SD 7.2) at 4 weeks, and 22.1 (SD7.4) at 30 weeks. The Treatment group had NLR of 19.6 (SD 8.9) at baseline, 27.6 (SD 9.1) at 4 weeks, and 27.8 (SD 8.4) at 30 weeks. The change in NLR from baseline in the Treatment compared to the Sham control group was 7.7 (95% CI 5.7, 9.7, p < 0.001) at 4 weeks and 10.4 (95% CI 7.8, 13.1, p < 0.001) at 30 weeks. There were similar benefits in CS.

**Conclusions:**

This pilot study of transpalpebral microcurrent demonstrated improved visual measures and is very encouraging as a potential treatment for dry AMD.

*Trial Registration*: NCT02540148, ClinicalTrials.gov.

**Supplementary Information:**

The online version contains supplementary material available at 10.1186/s40942-023-00471-y.

Age-related macular degeneration (AMD) is responsible for 8.7% of all blindness worldwide and is the most common cause of blindness in developed countries [1]. Its prevalence increases with age and is therefore magnified by population ageing [1]. Dry or atrophic AMD accounts for 85% of cases and is characterized by retinal pigmented epithelium dysfunction, and is a risk factor for, or even a precursor state of wet AMD, characterized by choroidal neovascularization [2, 3]. Geographic atrophy with loss of the retinal pigmented epithelium is accompanied by atrophy of adjacent photoreceptors and is a late stage of AMD [4]. Dry AMD is debilitating with loss of ability to read, recognize faces, see signs while driving, producing greater life stress, lower activity levels, greater risk of depression, functional disability and also an associated increased risk of cognitive impairment [5]. Risk factors for dry AMD include smoking, increasing age, higher serum cholesterol levels, and obesity [2]. While treatment with vascular endothelial growth factor (VEGF) inhibitors is effective in wet AMD, presently there are no approved treatments for dry AMD [5].

Microcurrent provides electrical stimulation to nerve fibres through cutaneous electrodes, using lower current than transcutaneous electrical nerve stimulation (TENS) [6]. Microcurrent is best known for skin healing with reduction of inflammation, improved local blood circulation, and improved mitochondrial function [7], and has even recently been adapted for direct cardiac application to improve reduced ejection fraction heart failure [8]. Wound healing is improved by microcurrent energy through alterations in cell metabolism, changes in extracellular matrix and proinflammatory signals [9]. Animal studies of transpalpebral electrical stimulation demonstrated a positive signal, preventing photoreceptor loss and improving retinal function [10], as well as stimulating Müller cells toward neuroregeneration and repair [11]. A study of human volunteers found a microcurrent effect on ganglion cells [12] that was polarity-dependent [13]. In twenty-eight patients with planned vitrectomy, thirteen received microcurrent pre-operatively. In treatment patients, positive effects on retinal cell function and survival, and reduced proinflammatory cytokines (IL-6, IL-8), and reduced bioactive lipid mediator expression (lysophosphatidylcholine), compared to 15 patients who did not receive microcurrent [14]. An early study demonstrated that transpalpebral microstimulation could be safely administered to patients with dry AMD, treating 25 eyes in seventeen patients receiving two to ten weekly treatments, finding improvement in visual acuity in 52% of eyes, but deteriorations in 26% [15].

Microcurrent stimulation for AMD was reviewed in 2017 by the National Institute for Health Research, with only a single placebo randomized study completed [6]. Recent reviews of microcurrent reviewing the same literature, concluded that electrical stimulation was promising for AMD [10, 16]. The randomized study included 22 patients given five days of microcurrent or placebo, finding visual acuity improvement of 5 or more ETDRS letters after one week in treated patients and none in the placebo group [17]. Two recent reports on subjects with low vision from a variety of pathologies found improvement in vision measures with microcurrent treatments over ten days [18, 19]. We conducted a trial of transpalpebral microcurrent using the MacuMira^™^ device compared to a sham control for patients with dry AMD. The main outcome was change in ETDRS visual acuity and contrast sensitivity measures from baseline, and differences in these changes between treated and untreated participants.

## Methods

### Trial design

This was a randomized parallel study with allocation 3:1 for treatment compared to Sham control (ethical approval by the Western Institutional Review Board, study number 1260150). Patients with dry AMD had a complete ophthalmic evaluation including ETDRS visual acuity testing before external micro current electrical stimulation with the MacuMira^™^. Initially, the protocol called for evaluation of participant’s eyes individually, treating both eyes if both had dry AMD. The protocol was changed on the advice of Health Canada to include in the analysis only one eye chosen randomly from each subject. Reporting of the study followed the 2010 CONSORT guidance [20]. To accommodate the pandemic, adjustments were made to accommodate changing rules about social distancing, PPI use, and stay-at-home orders. The extenuating circumstances from the COVID-19 pandemic were addressed using the CONSERVE 2021 statement [21].

### Participants

Participants were men and women with confirmed dry AMD, age fifty years of age or older, with visual loss and best-corrected vision 20/50 to 20/200, able to understand and comply with the requirements of the study and returning for all required visits. Participants were excluded if they were in general poor health, had actively treated cancer, had retinal pathology due to other causes, had a previous intravitreal injection or vitreo-retinal surgery, a history of seizure disorders, a dense cataract, or eyelid pathology at treatment sites. Also, patients with previous micro-stimulation treatment to the eyes, or glaucoma with a visual field defect of greater than 10 dB on the Humphrey visual field testing were excluded. (Additional file 1: Table S1).

The clinical trial took place at a single Image Optometry clinic in Coquitlam, British Columbia, Canada. Image Optometry is a community-based Optometry chain in British Columbia. All intake, consent and treatment took place at the same location. Participants were recruited from eye care professionals in and around the Coquitlam area aware of the basic inclusion criteria and through local community advertising. On referral, participants had additional screening by the primary investigator (KP). Participants were all ambulatory and recruited directly through newspaper and radio ads had initial screening asking about an existing diagnosis of dry AMD, previous eye injections, ability to drive, read, and recognize faces from across a room before a screening appointment with the PI. The screening process confirmed Dry AMD and ensured participants met inclusion and exclusion criteria. The study coordinator completed the case report form including demographics, ocular history, medical therapies, and supplements, and reviewed the inclusion and exclusion criteria. Participants were compensated up to $10 (CDN) for travel. Participants completed visual acuity and contrast sensitivity testing, and if VA was 20/50–20/200, they were invited to complete Optical Coherence Tomography, refraction and eye glass measurements and had their eyes dilated for an ocular exam or slit-lamp examination by the PI. (Additional file 1: Table S2) Participants then took home consent forms to review with family members, and their other health care providers. On each subsequent visit, participants were asked not to have face cream or make-up, and to have at least one glass of water prior to the visit. At the first appointment, the consent form was reviewed in full by the study coordinator. After signing the consent, the study protocol and procedures were explained again including accommodations required due to the pandemic.

Because of the pandemic, social distancing and PPI protection was followed for all visits. Participants were phoned before study visits by the study coordinator to discuss their ongoing interest in continuing with the study, transportation, and updated accommodations due to the pandemic, scheduling and other participant’s needs as they arose.

### Interventions

Participants were provided a comfortable and professional atmosphere, answered any questions, or concerns on the first visit, and on each visit provided time to visit the restroom prior to the assessment and treatment. The following are the steps for carrying out the treatment: 1. Electrodes placed on patient’s temples. 2. Demonstration of how head band and goggles fitted snugly, over eyes. Participant placed in reclining position and asked to keep eyes closed. 3. Gel applied to upper and lower eyelids. 4. Goggles connected to head band. 5. Electrodes connected to goggles. 6. Coordinator monitors participant positioning during entire procedure and enquires about what patient is experiencing.

The MacuMira System I (MacuMira Medical Devices Inc., Palm Desert, California, Canadian device class 2 licence 108571) is powered by alternating current, generating a pulsed waveform with a default varying frequency pattern from 1 to 50 Hz, and a current level between 50 and 200 μA. The signal is transmitted to the eye (through closed eyelids) by eyecup-shaped electrodes, connected to a headset. Signal parameters, timing, and auditory feedback control operations are controlled by a microprocessor housed in the control unit.

**Fig. 1:**
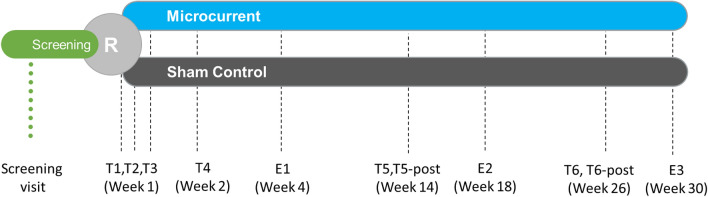
Study flow diagram

**Fig. 2:**
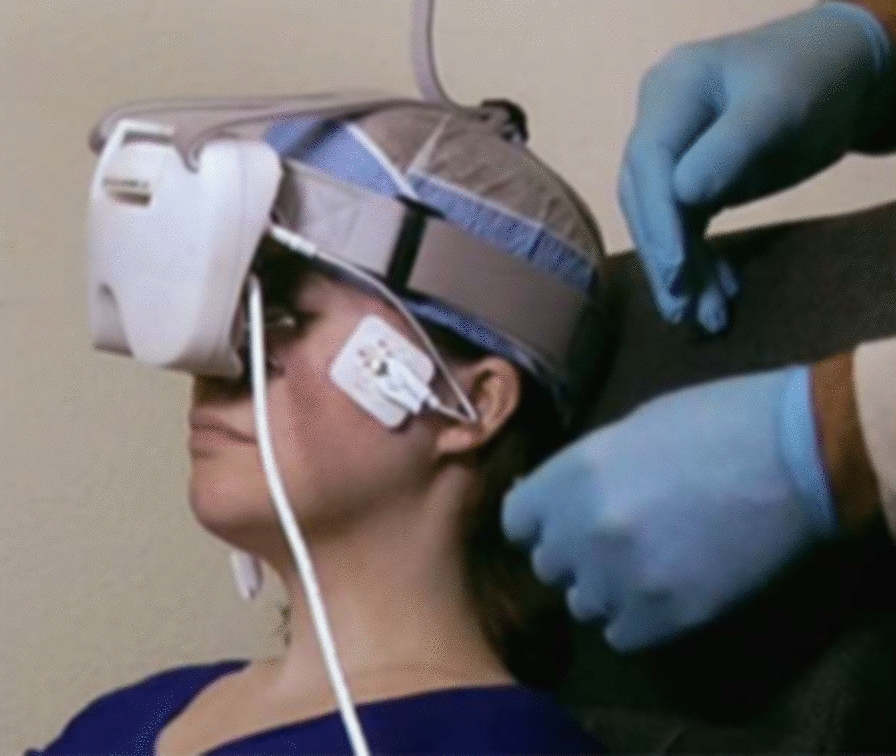
Microcurrent device

All participants received treatments (real or sham) on one, or both eyes if both eyes met inclusion criteria, for three days during the first week, then a single treatment during weeks 2, 14 and 26. (see Fig. 1) The treatment session duration was 30 min. The setup for the Treatment group had the MacuMira device plugged into the wall, while for the sham setup, a battery-operated device with signal lights (such as the on/off signal) with no current directed over the eye was used. (see Fig. 2) These parameters were derived at least in part from the medical literature on the subject [15, 22, 23].

The main outcome measures were Early Treatment of Diabetic Retinopathy Study (ETDRS) best corrected visual (VA) acuity converted to number of letters read (NLR) and Contrast Sensitivity (CS). Number of letters read (NLR) as well as contrast sensitivity (CS) were measured at 11 time points: T1-T4 (measured just before each of the first 4 treatments, completed within 2 weeks), E1 (evaluation without treatment 4 weeks after T1), T5 (just before 5th treatment 14 weeks after T1), T5-post (immediately after 5th treatment), E2 (evaluation without treatment 4 weeks after T5), T6 (just before 6th treatment 26 weeks after T1), T6-post (immediately after 6th treatment), and E3 (evaluation without treatment 4 weeks after T6). (See Fig. 1).

The main outcomes were NLR and CS measurements in the treatment group compared to the sham control group from baseline to evaluation one (E1) 4 weeks after the first treatment and evaluation 3 (E3) 4 weeks after the sixth and final treatment at week 26. Evaluation E1 followed four treatments over two weeks (three in the first week, one in the second week), and E3 followed four weeks after treatments five and six at week 14 and week 26 after the first treatment.

### Sample size

The groupwise standard deviations (SD) of the T1-E3 changes in NLR were 5.3 and 3.3 for the treatment and sham control groups respectively. Conservatively assuming the larger SD, and the groupwise sample sizes of 43 treatment subjects and 19 sham control, we had > 99% power to detect a true difference in delta NLR between groups of at least 8 (the primary hypothesis), via 2-sided test at alpha = 0.05. For smaller differences, we were also adequately powered for differences of 4, 5, 6, or 7, (76.9%, 92.1%, 98.1%, 99.7% respectively).

### Randomisation

With knowledge of how many participants were scheduled for their first visit, the PI contacted the study coordinator to ensure that participants were randomized in a ratio of three to one overall. Due to the onset of the pandemic and the need for social distancing only one participant could receive therapy at a time in the clinic. To schedule sufficient participants required a reduction of set-up time leading the study coordinator to put the morning patients into the treatment arm, switching the equipment over to sham control treatment for the rest of the participants that day.

### Statistical methods

Original data included measurements performed on 79 eyes within 62 patients. Using the ranuni [24] function within the statistical package SAS version 9.4 (SAS Institute, Cary, NC), a single eye from all subjects with two treated eyes was randomly selected, hence the analysis dataset contained a single record per subject. The total analysis sample size of N = 62 was comprised of 43 treatment and 19 sham control participants. To explore the possibility that bilateral patients may respond differently to treatment than unilateral patients, as a post-hoc sensitivity analysis we performed the original analysis again but restricted to those with bilateral treatment. This subset included 2 sham control subjects and 15 active treatment subjects (each with two records).

Descriptive statistics were computed and comparisons between groups made on age and sex. Comparisons were made via Wilcoxon rank-sum test (age) and chi-square test (sex). Comparisons on NLR or CS over time within and between groups were made using mixed effects repeated measures analysis of variance (RM-ANOVA), with contrasts estimated via least squares means. Two sets of contrasts were computed: cross-sectional between-group contrasts at each time point, and longitudinal change contrasts at each time point vs. T1 (within group), as well as between-group contrasts comparing within-group changes over time. The RM-ANOVA used 9 time points (excluded were the T5-post and T6-post due to potential temporary effects not pertinent to the current study of longer-term efficacy). Model fit was assessed via normal quantile–quantile plots of the standardized residuals. Analyses were performed using SAS version 9.4 (SAS Institute Inc., Cary, NC, USA).

The study was approved by the Western Institutional Review Board (study number 1260150) and was carried out according to the principles of Good Clinical Practice, the Declaration of Helsinki and the Tri-Council Policy Statement on ethical conduct for research involving humans [25, 26].

## Results

**Fig. 3:**
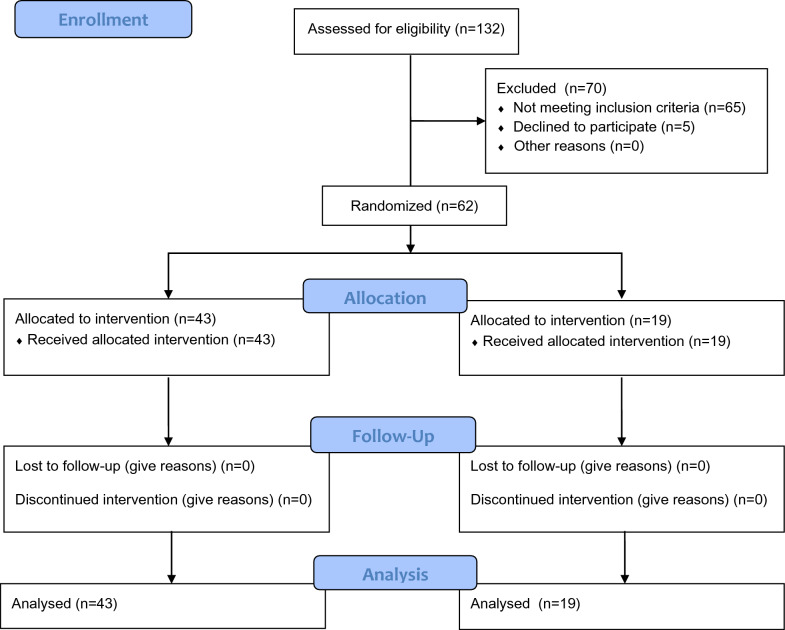
Consort flow diagram

A total of 132 subjects were screened and 67 subjects were consented to participate in the study. Of these, 5 subjects were withdrawn due to personal reasons associated with the COVID-19 pandemic. This left 62 subjects (38 female and 24 male) (see Fig. 3). The first participant entered the study December 2019, and the final participant completed the final evaluation October 2022. There were 43 subjects in the treatment group and 19 in the sham Control group and one eye only was included for analysis from each participant. See Table 1 for breakdown of demographics and baseline clinical characteristics.

**Table 1:** Baseline data

Parameters	Sham control n = 19	MicroCurrent n = 43	p value
Age years, (SD)	81.1 (6.1)	78.2 (6.5)	0.120
Female no. (%)	13 (68.4)	25 (58.1)	0.444
White race, no. (%)	16 (84.2)	39 (90.7)	0.457
Diabetes status (%)	2 (10.5)	7 (16.3)	0.553
Cataracts (%)	11 (57.9)	25 (58.1)	0.986
Hypertension (%)	6 (31.6)	20 (46.5)	0.272

Sex did not significantly differ between groups, with 58.1% and 68.4% female respectively in treatment and sham control groups (chi-square test p-value 0.444). Age did not significantly differ between groups, with mean (SD) 78.2 (6.5) in treatment and 81.1 (6.1) in sham control (Wilcoxon rank-sum test p-value 0.120).

Table 2 lists the mean NLR over time by group, with RM-ANOVA contrasts (with confidence intervals [CIs] and p-values testing differences between groups). At T1, the baseline measure, the treatment group started with a just-significantly lower mean NLR, with treatment–sham control contrast − 4.6 (95% CI − 9.1, − 0.0; p = 0.048). At each subsequent evaluation the direction of the difference between groups became positive and by evaluation E3, attained statistical significance favoring the treatment group with contrast 5.8 (1.4, 10.3; 0.011), while the sham control group declined modestly over time. The point estimates within treatment group increased from T1 with mean (SD) 19.6 (8.9) to E3 with mean (SD) 27.8 (8.4). The point estimates within the sham control group decreased from T1 with mean (SD) 24.2 (7.1) to E3 with mean (SD) 22.1 (7.4).

**Table 2:** Cross sectional comparisons of NLR by group

Time point	Treatment group mean (SD)	Sham control group mean (SD)	Treatment-Sham control difference (95% CI)*	Treatment-Sham control P-value (H0: difference = 0)*
T1	19.6 (8.9)	24.2 (7.1)	− 4.6 (− 9.1, − 0.0)	0.048
T2	24.0 (8.7)	23.7 (7.1)	0.3 (− 4.2, 4.8)	0.891
T3	26.3 (8.8)	24.1 (6.6)	2.2 (− 2.2, 6.7)	0.318
T4	27.4 (8.9)	24.1 (6.6)	3.4 (− 1.1, 7.9)	0.138
E1	27.6 (9.1)	24.2 (7.2)	3.1 (− 1.6, 7.8)	0.189
T5	26.4 (8.2)	23.3 (6.6)	3.0 (− 1.3, 7.2)	0.169
E2	26.2 (8.4)	21.7 (7.6)	3.8 (− 0.6, 8.2)	0.089
T6	26.0 (8.1)	22.3 (7.1)	3.8 (− 0.5, 8.1)	0.080
E3	27.8 (8.4)	22.1 (7.4)	5.8 (1.4, 10.3)	0.011

* Contrasts and p-values computed via RM-ANOVA

Table 3 lists the changes in NLR vs. T1 over time by group, with RM-ANOVA contrasts (with CIs and p-values testing differences between groups). At every time point from T2 to E3, change in mean NLR significantly favored the Treatment group, with Treatment–Sham Control contrasts increasing nearly monotonically from 4.9 (3.6, 6.2; < 0.001) at T2-T1 to 10.4 (7.8, 13.1; < 0.001) at E3-T1.

**Table 3:** Mean changes in NLR vs. T1 by group

Time points	Treatment group contrast (95% CI)^a^	Sham control group contrast (95% CI)^a^	Treatment-Sham control contrast (95% CI)^a^	Treatment-Sham control P-value (H0: difference = 0)^a^
T2-T1	4.4 (3.7, 5.1)	− 0.5 (− 1.5, 0.6)	4.9 (3.6, 6.2)	< 0.001
T3-T1	6.7 (5.8, 7.5)	− 0.2 (− 1.4, 1.1)	6.8 (5.3, 8.4)	< 0.001
T4-T1	7.7 (6.7, 8.8)	− 0.2 (− 1.8, 1.3)	8.0 (6.1, 9.8)	< 0.001
E1-T1	7.6 (6.5, 8.7)	− 0.1 (− 1.8, 1.6)	7.7 (5.7, 9.7)	< 0.001
T5-T1	6.5 (5.4, 7.6)	− 1.0 (− 2.7, 0.6)	7.5 (5.6, 9.5)	< 0.001
E2-T1	7.2 (6.0, 8.4)	− 1.2 (− 3.0, 0.6)	8.4 (6.3, 10.5)	< 0.001
T6-T1	6.3 (5.1, 7.6)	− 2.0 (− 4.0, − 0.1)	8.4 (6.1, 10.7)	< 0.001
E3-T1	8.2 (6.7, 9.7)	− 2.2 (− 4.4, 0.0)	10.4 (7.8, 13.1)	< 0.001

^a^Contrasts and p-values computed via RM-ANOVA

Table 4 lists the mean CS over time by group, with RM-ANOVA contrasts (with CIs and p-values testing differences between groups). At T1, mean CS only borderline significantly favored the Sham Control group, with Treatment–Sham Control contrast − 0.12 (− 0.24, 0.01; 0.062). The direction of the difference between groups changes at T3, and eventually attaining statistical significance favoring the Treatment group at T6 and E3, with E3 contrast 0.13 (0.01, 0.25; 0.034).

**Table 4:** Cross sectional comparisons of CS by group

Time point	Treatment group mean (SD)	Sham control group mean (SD)	Treatment-Sham control contrast (95% CI)^a^	Treatment-Sham control P-value (H0: difference = 0)^a^
T1	1.00 (0.24)	1.11 (0.17)	− 0.12 (− 0.24, 0.01)	0.062
T2	1.13 (0.22)	1.14 (0.20)	− 0.01 (− 0.12, 0.10)	0.860
T3	1.19 (0.21)	1.14 (0.21)	0.06 (− 0.06, 0.17)	0.337
T4	1.23 (0.22)	1.16 (0.22)	0.08 (− 0.04, 0.20)	0.179
E1	1.20 (0.20)	1.18 (0.26)	0.03 (− 0.09, 0.14)	0.661
T5	1.20 (0.22)	1.13 (0.25)	0.06 (− 0.06, 0.18)	0.332
E2	1.23 (0.20)	1.09 (0.26)	0.10 (− 0.02, 0.22)	0.112
T6	1.24 (0.21)	1.08 (0.22)	0.16 (0.04, 0.28)	0.009
E3	1.24 (0.21)	1.11 (0.24)	0.13 (0.01, 0.25)	0.034

^a^Contrasts and p-values computed via RM-ANOVA

Table 5 lists the changes in CS vs. T1 over time by group, with RM-ANOVA contrasts (with CIs and p-values testing differences between groups). At every time point from T2 to E3, change in mean CS significantly favors the treatment group, with treatment–sham control contrasts increasing from 0.11 (0.04, 0.17; 0.002) at T2-T1 to 0.25 (0.16, 0.34; < 0.001) at E3-T1.

**Table 5:** Mean changes in CS vs. T1 by group

Time points	Treatment group contrast (95% CI)^a^	Sham control group contrast (95% CI)^a^	Treatment-Sham control contrast (95% CI)^a^	Treatment-Sham control P-value (H0: difference = 0)^a^
T2-T1	0.13 (0.09, 0.16)	0.02 (− 0.03, 0.08)	0.11 (0.04, 0.17)	0.002
T3-T1	0.20 (0.15, 0.24)	0.02 (− 0.04, 0.09)	0.17 (0.09, 0.25)	< 0.001
T4-T1	0.24 (0.20, 0.28)	0.04 (− 0.02, 0.10)	0.20 (0.12, 0.27)	< 0.001
E1-T1	0.20 (0.15, 0.25)	0.06 (− 0.02, 0.13)	0.14 (0.05, 0.23)	0.002
T5-T1	0.19 (0.14, 0.24)	0.02 (− 0.06, 0.10)	0.18 (0.08, 0.27)	0.001
E2-T1	0.20 (0.15, 0.25)	− 0.02 (− 0.09, 0.06)	0.21 (0.13, 0.30)	< 0.001
T6-T1	0.24 (0.19, 0.29)	− 0.03 (− 0.11, 0.04)	0.28 (0.19, 0.37)	< 0.001
E3-T1	0.24 (0.19, 0.29)	− 0.01 (− 0.08, 0.06)	0.25 (0.16, 0.34)	< 0.001

^a^Contrasts and p-values computed via RM-ANOVA

In sensitivity analyses restricted to those with bilateral treatment, results within the active treatment were of interest. Statistically significant improvements in NLR of over 7 from baseline visit to subsequent time points including the study end point were found, as well as statistically significant improvements in CS from baseline visit to subsequent time points including the study end point.

There were no device-related adverse events, anticipated or unanticipated, identified during the study. There were no cross overs from the sham control group.

### Discussion

This study reports on the largest number of patients participating in a controlled trial of microcurrent for dry AMD. The sham control group at baseline has better vision than the treatment group with number of letters read starting at 24.2 on the first visit, declining to 22 at the last measurement approximately 30 weeks later. In contrast, treatment participants receiving active therapy with micro-current had lower ETDRS visual acuity assessment of number of letters read at baseline with a score of 19.6, and a rapid rise peaking four weeks after the first visit, maintained at the study end (E3). During the first week, treatment patients received three treatments and in the second week, a fourth treatment (Table 3). It is notable that despite the challenges of the pandemic, participants returned for their therapy.

This study adds to a growing literature on the role of microcurrent for AMD. Microcurrent stimulation for AMD was reviewed in 2017 by the National Institute for Health Research, with only a single placebo randomized study completed [6]. The randomized study included 22 patients given five days of microcurrent or placebo, finding visual acuity improvement of 5 or more ETDRS letters after one week in treated patients and none in the placebo group [17]. More recent reviews of microcurrent concluded that electrical stimulation was promising for AMD [10, 16]. Following these reviews, two reports on subjects with low vision from a variety of pathologies receiving microcurrent treatments over ten days found improvements in vision measures [18, 19].

Nutritional therapy has been a mainstay for dry AMD, including lutein and zeaxanthin with an 18% slowing of progression to advanced AMD in AREDS2 [5]. Other therapies for dry AMD have tried anti-inflammatory and neuroprotective agents, vasodilators, and complement inhibitors, to slow progression to geographic atrophy [2, 5]. While a phase II study of monthly injections of pegcetacoplan over 18 months, targeting complement factors C3 and C5, found a 29% (95% CI 9–49) reduction of new geographic atrophy, there were more episodes of endophthalmitis [27]. Lampalizumab targeting Factor D in the alternative complement pathway showed early promise [28], but phase 3 trials were negative [29]. Mitochondrial senescence from mitochondrial DNA damage, reduced ATP production, and altered cellular autophagy provide other targets of therapy for the highly metabolic retinal pigment epithelia, all potentially impacted by microcurrent [30].

Loss of visual acuity is used as the main outcome in many clinical studies [31] including a UK national database study of treatment-naïve eyes with wet AMD treated with a an anti-vascular endothelial growth factor antibody with follow-up over three years [32]. After 93,000 injections in 11,000 patients with baseline visual acuity of 55 (Snellen equivalent acuity of 6/21), at one year there was an improvement by 2 letters to 57, and after 2 years remained improved at 56 letters, falling to 53 letters after 3 years [5, 32]. This contrasts with the change found in this study in the treatment group over 30 weeks, increasing from 19.6 to 27.8 letters.

### Limitations

The impact of the pandemic and need for social distancing on the randomization in this study increases the potential for bias, and the confidence in the results is therefore diminished by it. The sustained improvement in the treatment group, and three to one enrollment, compared to the sham control group which had a slow decline in vision measure over the study, make regression to the mean less likely, as well as similar finding with CS.

The study’s community setting is a strength with referral of participants from local health care providers and through advertisements, making the study population generalizable to the population in this region.

### Interpretation

This controlled study of microcurrent for dry AMD found visual improvement in the treatment group. This data provides cautious optimism that microcurrent can improve vision, at least over 30 weeks for dry AMD. While very encouraging, further studies are needed to replicated these results and to assess how to maximize the treatment benefit of microcurrent for dry AMD.

## Supplementary Information

**Additional file 1: Table S1** Online Supplement. Inclusion/Exclusion criteria for enrolled participants. **Table S2** Online Supplement. Measurement of visual acuity.

## Data Availability

The datasets used and/or analysed during the current study are available from the corresponding author on reasonable request.

## References

[1] Wong WL, Su X, Li X, Cheung CMG, Klein R, Cheng C-Y (2014). Global prevalence of age-related macular degeneration and disease burden projection for 2020 and 2040: a systematic review and meta-analysis. Lancet Glob Health.

[2] Schultz NM, Bhardwaj S, Barclay C, Gaspar L, Schwartz J (2021). Global burden of dry age-related macular degeneration: a targeted literature review. Clin Ther.

[3] Ambati J, Fowler BJ (2012). Mechanisms of age-related macular degeneration. Neuron.

[4] Keenan TD, Agrón E, Domalpally A, Clemons TE, van Asten F, Wong WT (2018). Progression of geographic atrophy in age-related macular degeneration: AREDS2 report number 16. Ophthalmology.

[5] Mitchell P, Liew G, Gopinath B, Wong TY (2018). Age-related macular degeneration. Lancet.

[6] Waugh N, Loveman E, Colquitt J, Royle P, Yeong JL, Hoad G (2018). Treatments for dry age-related macular degeneration and Stargardt disease: a systematic review. Health Technol Assess.

[7] Yu C, Hu Z-Q, Peng R-Y (2014). Effects and mechanisms of a microcurrent dressing on skin wound healing: a review. Mil Med Res.

[8] Schmitto JD, Napp LC, Mariani S, Hanke JS, Li T, Vogel-Claussen J (2022). First-in-man implantation of a cardiac microcurrent device for chronic systolic heart failure. ASAIO J.

[9] Khan I, Arany P (2015). Biophysical approaches for oral wound healing: emphasis on photobiomodulation. Adv Wound Care.

[10] Liu J, Tong K, Lin Y, Lee VW, So KF, Shih KC (2021). Effectiveness of microcurrent stimulation in preserving retinal function of blind leading retinal degeneration and optic neuropathy: a systematic review. Neuromodul Technol Neural Interface.

[11] Enayati S, Chang K, Achour H, Cho K-S, Xu F, Guo S (2020). Electrical stimulation induces retinal Müller cell proliferation and their progenitor cell potential. Cells.

[12] Blum M-C, Solf B, Hunold A, Klee S (2021). Effects of ocular direct current stimulation on full field electroretinogram. Front Neurosci.

[13] Blum M-C, Hunold A, Solf B, Klee S (2021). Ocular direct current stimulation affects retinal ganglion cells. Sci Rep.

[14] Colombo L, Caretti A, Dei Cas M, Luciano F, Romano D, Paroni R (2021). Vitreous composition modification after transpalpebral electrical stimulation of the eye: biochemical analysis. Exp Eye Res.

[15] Chaikin L, Kashiwa K, Bennet M, Papastergiou G, Gregory W (2015). Microcurrent stimulation in the treatment of dry and wet macular degeneration. Clin Ophthalmol.

[16] Sanie-Jahromi F, Azizi A, Shariat S, Johari M (2021). Effect of electrical stimulation on ocular cells: a means for improving ocular tissue engineering and treatments of eye diseases. BioMed Res Int.

[17] Anastassiou G, Schneegans A-L, Selbach M, Kremmer S (2013). Transpalpebral electrotherapy for dry age-related macular degeneration (AMD): an exploratory trial. Restor Neurol Neurosci.

[18] Livengood H, Wollstein G, Ishikawa H, Wu M, Liu M, Achanta P (2021). Preliminary results of repetitive transorbital alternating current stimulation in optic neuropathies. Investig Ophthalmol Vis Sci.

[19] Granata G, Falsini B (2022). Preliminary results of transorbital alternating current stimulation in chronic low vision: correlation of clinical and neurophysiological results. Neuromodulation.

[20] Schulz KF, Altman DG, Moher D (2010). CONSORT 2010 Statement: updated guidelines for reporting parallel group randomised trials. BMC Med.

[21] Orkin AM, Gill PJ, Ghersi D, Campbell L, Sugarman J, Emsley R (2021). Guidelines for reporting trial protocols and completed trials modified due to the COVID-19 pandemic and other extenuating circumstances: the CONSERVE 2021 statement. JAMA.

[22] Sehic A, Guo S, Cho K-S, Corraya RM, Chen DF, Utheim TP (2016). Electrical stimulation as a means for improving vision. Am J Pathol.

[23] Markowitz SN, Devenyi RG, Munk MR, Croissant CL, Tedford SE, Ruckert R (2020). A double-masked, randomized, sham-controlled, single-center study with photobiomodulation for the treatment of dry age-related macular degeneration. Retina.

[24] Mortensen LQ, Andresen K, Burcharth J, Pommergaard H-C, Rosenberg J (2019). Matching cases and controls using SAS^®^ software. Front Big Data.

[25] World Medical Association Declaration of Helsinki. JAMA. 2013;310(20):2191–2194. 10.1001/jama.2013.281053. 10.1001/jama.2013.281053 24141714

[26] Canadian Institutes of Health Research NSaERCoC, and Social Sciences and Humanities Research Council of Canada. Tri-Council Policy Statement. Ethical Conduct for Research Involving Humans. CIHR, NSERC, SSHRC of Canada; 2014.

[27] Liao DS, Grossi FV, El Mehdi D, Gerber MR, Brown DM, Heier JS (2020). Complement C3 inhibitor pegcetacoplan for geographic atrophy secondary to age-related macular degeneration: a randomized phase 2 trial. Ophthalmology.

[28] Yaspan BL, Williams DF, Holz FG, Regillo CD, Li Z, Dressen A (2017). Targeting factor D of the alternative complement pathway reduces geographic atrophy progression secondary to age-related macular degeneration. Sci Transl Med.

[29] Halawa OA, Lin JB, Miller JW, Vavvas DG (2021). A review of completed and ongoing complement inhibitor trials for geographic atrophy secondary to age-related macular degeneration. J Clin Med.

[30] Tong Y, Zhang Z, Wang S (2022). Role of mitochondria in retinal pigment epithelial aging and degeneration. Front Aging.

[31] Bellmann C, Unnebrink K, Rubin GS, Miller D, Holz FG (2003). Visual acuity and contrast sensitivity in patients with neovascular age-related macular degeneration. Graefes Arch Clin Exp Ophthalmol.

[32] Committee W (2014). The neovascular age-related macular degeneration database: multicenter study of 92,976 ranibizumab injections: report 1: visual acuity. Ophthalmology.

