# Supplementary material for: Evaluation of visual acuity in dry AMD patients after microcurrent electrical stimulation
Source: Int J Retina Vitreous. 2023 Jun 18;9:36. doi: 10.1186/s40942-023-00471-y (PMC10278316; doi:10.1186/s40942-023-00471-y)
Supplement: Supplementary file 1 — Additional file 1: Table S1 Online Supplement. Inclusion/Exclusion criteria for enrolled participants. Table S2 Online Supplement. Measurement of visual acuity. [file 40942_2023_471_MOESM1_ESM.docx]

| Inclusion Criteria | Exclusion Criteria |
| --- | --- |
| - Fifty years of age or older | - Any visually significant retinal pathology other than dry AMD |
| - Male or female | - Previous intravitreal injection |
| - Best-corrected vision 20/50 to 20/200 for each enrolled eye | - Seizure disorders |
| - Confirmed diagnosis of dry AMD | - Previous vitreo-retinal surgery. (ERM, PPV, RD) |
| - Vision loss attributable to dry AMD | - Dense cataract |
| - Subjects must be highly motivated, alert, oriented, mentally competent and able to understand and comply with the requirements of the study, abide by the restrictions, return for all required visits, and provide voluntary informed consent | - Eyelid pathology at the treatment sites |
|  | - Glaucoma patients with a visual field mean |
|  | - defect of greater than 10dB on Humphrey visual field testing |
|  | - Glasses are up to date (prescription <1 year old) |
|  | - Any prior electrical micro-stimulation treatment to the eyes |
|  | - Poor general health |
|  | - Active cancer |

Table S1. Online Supplement. Inclusion/Exclusion criteria for enrolled participants

| Measurement of visual acuity |
| --- |
| **ETDRS visual acuity:** Performed on each qualified eye of all subjects prior to treatment and evaluation on follow-up visits. It was also performed after treatments 5 and 6. Contrast sensitivity was also similarly performed. |
| **Illumination**: The same room and illumination were used for each patient. |
| **Glasses**: We ensured on the first visit that the participant’s glasses (if they brought them) were up to date (within 2 years) and then used the same glasses for every subsequent measurement. |
| VA measurement distance: VA was measured at 2 meters as per the specification of the ETDRS and the Contrast Sensitivity Chart used. |
| **VA measurement**: Participants occluded the left eye with an occluder, while standing behind the line placed on the floor and with their glasses on (if worn for distance), they would read down the charts as far as possible without coaxing using the right eye. This would then be repeated for the left eye. |
|  |

Table S2. Online Supplement. Measurement of visual acuity
